# Nemolizumab efficacy for severe sleep disturbance in pediatric atopic dermatitis with mild skin lesions

**DOI:** 10.1016/j.jdcr.2026.04.027

**Published:** 2026-04-24

**Authors:** Yuji Fujita, Manabu Miyamoto, Masaya Kato, Hideaki Shiraishi, Shigemi Yoshihara

**Affiliations:** a Department of Pediatrics, Dokkyo Medical University, Tochigi, Japan; b Allergy Center, Dokkyo Medical University Hospital, Tochigi, Japan

**Keywords:** AHEAD, atopic dermatitis, nemolizumab, pediatric patient, sleep disturbance

## Introduction

Atopic dermatitis (AD) can cause various symptoms, including itching, changes in skin appearance, and sleep disturbances. Moreover, it is an established risk factor for several comorbidities and exerts detrimental effects on physical and mental health.^1^ Sleep disturbance has recently attracted attention because of its adverse effects on mental health.^2^ Although topical treatment improves skin rashes, itching is often more difficult to address. Furthermore, when AD symptoms do not improve sufficiently, patients often experience disturbed sleep. Herein, we report 2 pediatric cases of AD in which nemolizumab effectively alleviated sleep disturbance associated with severe itching that persisted despite successful topical treatment.

### Case 1

The first case presents a 6-year-old female patient with AD who showed considerable improvement in the accompanying rash in response to topical treatment. However, severe itching persisted, particularly at night, disturbing sleep. The Eczema Area and Severity Index (EASI) score was 7.4 while the patient was receiving oral antihistamines and topical treatment (betamethasone butyrate propionate and delgocitinib), but it worsened with tapering of topical corticosteroids. The Patient-Oriented Eczema Measure (POEM) score was 23, and the sleep disturbance score was 4, indicating severe impairment in quality of life and sleep (Table I). Following subsequent treatment with nemolizumab (30 mg) administered subcutaneously at 4-wk intervals, the patient showed slight improvement in the EASI and a marked improvement in the POEM sleep disturbance score, which decreased from 4 to 0 after the first administration. The patient has since continued to sleep well.

**Table I:** Clinical severity scores

	Case 1 (6-y-old girl)			Case 2 (9-y-old girl)		
	Pretreatment	1 mo	6 mo	Pretreatment	1 mo	6 mo
EASI (0-72)	7.4	5.4	3	5.9	0.2	0.5
POEM (0-28)	23	5	8	25	0	1
POEM (sleep disturbance) (0-4)	4	0	0	4	0	0
PP-NRS (0-10)	8	2	4	8	0	0

*EASI,* Eczema Area and Severity Index; *POEM,* Patient-Oriented Eczema Measure; *PP-NRS,* Peak Pruritus Numerical Rating Scale.

### Case 2

This case presents a 9-year-old female patient with AD in whom the rash improved considerably with topical treatment; however, severe itching persisted, particularly at night, contributing to sleep disruption. The patient’s older sister had severe AD that was fully responsive to dupilumab. Therefore, a 3-m trial of dupilumab was initiated 1 y prior to starting nemolizumab. However, itching and sleep disturbance did not improve, and dupilumab was discontinued. At the time of nemolizumab initiation (30 mg administered every 4 wk), the EASI score was 5.9 while the patient was receiving oral antihistamines and topical treatment (betamethasone butyrate propionate and delgocitinib), with worsening observed during tapering of topical corticosteroids. The POEM score was 25, and the sleep disturbance score was 4, indicating severe impairment in quality of life and sleep (Table I). Following nemolizumab initiation, the patient showed slight improvement in the EASI score and a marked improvement in the POEM sleep disturbance score, which decreased from 4 to 0 after the first administration. The patient has since continued to sleep well.

## Discussion

Among the range of AD-associated symptoms, itching is the most commonly reported and troublesome, both in adult and pediatric patients. Even with relatively milder rashes, some patients with AD experience severe itching,^3^ and treatment in such cases should aim at more than merely alleviating the skin rash. Recently, the Aiming High in Eczema/Atopic Dermatitis approach has been proposed, combining treat-to-target principles and shared decision-making while highlighting the importance of patients’ subjective symptoms in AD management.^4^ Based on this approach, patients select 1-3 troublesome features associated with AD that they consider important. In our previous study on children treated using the Aiming High in Eczema/Atopic Dermatitis approach, multiple pediatric patients identified sleep disorders as troublesome features of AD,^5^ thereby emphasizing the need to address them to achieve true remission.

Sleep disorders reportedly exert detrimental effects on development and mental health, including depression, anxiety, and suicide,^6^ and these effects may begin at a young age.^7^ Moreover, pruritus-induced sleep disturbances in childhood AD are considered a risk factor for conduct and emotional problems as cumulative life-course impairments.^8^ These findings highlight the importance of adequately addressing childhood sleep disorders early in life. Nemolizumab, which targets interleukin (IL)-31, is typically used to treat AD-associated itching that is not fully responsive to topical treatments. It may produce rapid improvement in symptoms such as itch and sleep disturbance because IL-31 is a key neuroimmune mediator of itch; blockade of IL-31 receptor A interrupts itch signaling at the sensory neuron level, thereby improving pruritus and secondarily sleep before major changes in skin lesions become evident. In our cases, improvement in itching and sleep disturbance (POEM sleep disturbance score decreased from 4 to 0 at 1 m) was rapid.

Although clinical studies demonstrating the efficacy of nemolizumab have reported improvement in itching and sleep disorders in children with rash severity of EASI ≥10,^9^ its efficacy in mild AD (EASI <10) has not been studied. Nguyen et al reported 3 adult cases in which skin rash improved with dupilumab treatment, although itching and sleep disturbances did not sufficiently improve; nemolizumab was effective in treating these symptoms.^10^ In the present cases, although the rash improved with topical treatment, the patients continued to experience severe itching and sleep disturbance, and nemolizumab was highly effective in alleviating these symptoms. There are no established data on the optimal duration of biological agent use for any disease, including allergic conditions. However, if the goal is to maintain remission without relapse, long-term regular administration may be preferable to episodic use.

Assessing AD severity and indications for systemic treatment should not rely solely on rash severity but should also take into account symptoms such as itching and sleep disturbance. Sleep disorders, especially in childhood, may impose a significant cumulative lifetime burden; therefore, aggressive systemic treatment, particularly with nemolizumab, should be considered when sleep disturbances are refractory to topical treatment, even in cases with mild rash.

### Declaration of generative AI and AI-assisted technologies in the writing process

No AI or AI-assisted technologies were used in preparing this manuscript.

## Conflicts of interest

None disclosed.
